# Engaging Stakeholders, from Inception and Throughout the Study, is Good Research Practice to Promote use of Findings

**DOI:** 10.1007/s10461-019-02574-w

**Published:** 2019-07-03

**Authors:** Samuel Kalibala, Tara Nutley

**Affiliations:** 1 Palladium, Project SOAR, 4301 Connecticut Ave, Suite 280, Washington, DC 20008 USA; 2 Data, Informatics and Analytical Solutions, Palladium, 308 West Rosemary Street, Suite 203, Chapel Hill, NC 27517 USA

**Keywords:** Research utilization, Knowledge translation, Research advisory committees, Capacity strengthening, Stakeholder engagement, HIV operations research

## Abstract

The need for research-informed programming and policy making is well established. However, there is limited evidence that, when researchers actively promote utilization of research findings, stakeholders use such findings for decision making in low- and middle-income countries (LMIC). A common barrier for research uptake in LMIC is that researchers focus on passive dissemination of final findings as the primary vehicle to affect research uptake. A more active approach to facilitating research utilization (RU) is necessary. Project SOAR, a six-year USAID-funded operations research project, recognized this gap and developed an approach to include the end data users in the research process from inception to final results dissemination. In this commentary, we make recommendations for active facilitation of research uptake using emerging lessons from SOAR’s RU process that focuses on ongoing engagement of stakeholders throughout the life of the study.

## Introduction and Purpose

The need for research results to strengthen programming and policy making is well established [1]. With the increasing demand by donors and other stakeholders for evidence of the programmatic benefits of investments in research [2–4], research institutions are challenged to ensure that their research findings are considered by relevant stakeholders who make policy/program decisions [5].

To address this challenge, Project SOAR (Supporting Operational AIDS Research), a 6 year USAID-funded research project with over 60 activities in 21 countries, adopted a systematic research utilization (RU) approach equipped with a RU guide and tools [6] and a full-time knowledge use broker (RU Advisor) providing technical assistance to study teams to engage stakeholders from study inception throughout the study. In this paper, based on emerging lessons learned from strategically engaging stakeholders, we make recommendations to research institutions on how to ensure that decision-makers consider their research to inform policies and programs.

The aim of this commentary is to contribute to the further understanding of the link between active encouragement and support of RU and, the actual use of research results. To guide this exploration, we draw lessons from SOAR’s process and achievements in systematically facilitating its researchers to integrate RU into the research process. We use data from activity briefs^1^ and results briefs,^2^ supplemented by study progress reports and e-mails from investigators, to illustrate these lessons. As of the date of this publication, many of SOAR’s studies were still in the implementation stage; thus, the lessons we present mainly represent RU experiences from study inception to data interpretation of preliminary findings with a limited number of final study findings. Sharing these early lessons, without waiting for final study results, is critical for promptly informing and strengthening the RU process in SOAR and other research programs. Moreover, highlighting the early added value of the process with those implementing it strengthens their commitment to and potential sustainability of RU implementation.

### Brief Overview of SOAR’s Research Utilization Process

SOAR’s RU process begins with the identification and engagement of stakeholders who provide input into study design to ensure that researchers design studies that answer questions relevant to local programs and policies. It continues with engaging stakeholders during the implementation of the study, the analysis of data, and the planning to translate findings into action, all supported by standardized RU guidance. RU also occurs at the end of the study when stakeholders review the findings and develop recommendations to change policy or programs. To support its researchers in implementing the RU process, SOAR has embraced the principle of a knowledge broker [7, 8] by designating the position of RU Advisor on its management team. The RU Advisor provides technical support to study teams by disseminating the RU guide and tools to investigators and stakeholders; ensuring RU is integrated in the study protocol; and participating in the identification of key stakeholders and in the preparation and conduct of meetings with stakeholders. The RU Advisor provides this support through e-mails, phone calls, webinars, and country visits from study inception throughout implementation and dissemination of preliminary and final findings.

## Lessons Learned and Recommendations

### Assessing the Stakeholder and Policy Landscape is Key to RU Success

The mechanism through which research influences policy and program change is not linear [9–11]. Stakeholders respond to multiple factors, not just the evidence from studies, to make decisions. Translating research findings into action thus requires assessment of the research-and-policy/program dynamics within which the study is being conducted [8]. In addition, lack of timeliness or opportunity to use the research is one of the most prominent barriers revealed in a meta-analysis of RU experiences [12]. Hence, we recommend that efforts to encourage RU should seek to understand the times and opportunities for influencing policy/program change together with contextual factors that may influence decision making. For example, if the research was aimed to influence a national HIV prevention of mother-to-child transmission (PMTCT) plan, the researcher should be aware of the cycle for review and revision of the plan, and any outstanding policy/program gaps so that they can design their study to be relevant and target the right moment to engage key decisionmakers with results of their study.

SOAR researchers conduct a stakeholder and policy assessment, together with in-country co-investigators, during scouting visits to study countries at the study protocol development stage to understand contextual factors that influence decision-making. SOAR’s RU guide comes with a tool named *Linking Data with Questions and Decisions* that SOAR researchers use to highlight the information that the study intends to produce that may influence programmatic and/or policy decisions and the specific decision that study findings can influence; and the stakeholders that can make and/or implement the decision that the findings support. The researchers also use another tool, the *Stakeholder Engagement Matrix*, to identify stakeholders to be engaged, including their levels of influence, and specify the mode and dates of engagement for each key stakeholder throughout the life of the study. Also recorded are the issues that stakeholders raise about study design and methodology and the commitments they make to support the study. It also includes a follow-up column, that researchers fill out after each engagement, recording how they addressed the issues and whether the stakeholders met their commitments. Researchers initially use this information to inform the RU section of SOAR’s research protocol, in which they state how they have so far engaged stakeholders in the study design, and how they propose to continue engaging stakeholders throughout the study. Thus, through this process, the researchers not only get to learn the landscape of stakeholders and contextual factors that might influence decision-making, they devise how to address these issues in the study design.

### Research Advisory Committees or Technical Working Groups are Vital Forums for Engaging Stakeholders

Engagement ensures policy-relevant research by enabling researchers to consider the needs and inputs of stakeholders into research questions; and research-based policy by enabling stakeholders to trust, access, and apply research evidence [8]. As part of the scouting visit, before starting a study, SOAR researchers assess the existence of a forum for engaging stakeholders. For some SOAR studies researchers identified pre-existing technical working groups (TWGs)—such as a pediatric AIDS TWG—that they used effectively as forums for engaging stakeholders, rather than creating a study-specific research advisory committee (RAC). In most SOAR studies, however, it was necessary for the researchers to form study-specific RACs. To establish the RAC, they used the tool, *Advisory Panel Terms of Reference (ToR) Checklist*, from the RU guide, to facilitate agreement of RAC members on their modus operandi and elect a chair. The most common stakeholders engaged are National AIDS Councils (NAC) and the HIV program in the Ministries of Health (MOH) at the national and sub-national levels. Usually, national level stakeholders develop policies and program guidelines while sub-national level stakeholders make operational decisions for implementing these programs. In addition to the HIV program, researchers have engaged appropriate sections of the government for the topic being studied. For example, a study on the family planning needs of people living with HIV (PLHIV) in Tanzania [13] engaged the reproductive health division of the MOH in addition to the HIV program. SOAR researchers have also engaged donors and providers of services that are under investigation, who are crucial for taking up findings to improve these services. In addition, researchers engaged service beneficiaries such as young people. For example, the RAC of the Zambia Project YES! study [14] includes young people living with HIV (YPLHIV). In the RAC or TWG meetings, SOAR researchers present study objectives and methodologies using PowerPoint and activity briefs; and stakeholders ask questions for clarification and give input into the study design as well as practical aspects of how to engage members of the study population in data collection. For example, prior to a survey among HIV-positive orphans and other vulnerable children (OVC) in Zambia [15], RAC members proposed to the investigators that community-based counselors, supporting the OVC, be oriented to the study so that they can educate and counsel the OVC and their caregivers, about the study, before data collectors approached the homes. Using the above approach, SOAR study teams have successfully engaged stakeholders on an ongoing basis from the inception of studies onwards.

### Stakeholder Engagement is an Active Process of Mutual Accountability

Continuous engagement of stakeholders from the beginning and throughout the study is a practice that is promoted widely [16, 17] and important because results uptake can occur while a study is still ongoing [5]. To ensure local ownership of results, investigators should always engage stakeholders in study design whether writing a research proposal to raise funds, or a study protocol for research that is already funded. Stakeholders can use findings from baseline and formative surveys to address program gaps while the main study is ongoing. In a SOAR study to strengthen PMTCT services in Lesotho using community health workers (CHW) [18], a baseline survey showed that health facilities in one district had insufficient CHWs to trace PMTCT mothers. Upon receiving these findings, the district health management team recruited, trained, and deployed 46 CHW. Further, if the study has multiple rounds of data collection, stakeholders can use data from subsequent phases of the study to assess ongoing improvement comparable to a continuous quality improvement (CQI) process [19] or continuous learning and adaptive management (CLA) [20].

When SOAR researchers present their study methodology to stakeholders, they learn about stakeholder concerns and receive context-specific input and ensure that they respond to these concerns in a timely manner. For example, when SOAR investigators presented a study design strengthening PMTCT and antiretroviral (ARV) adherence in eSwatini using a wholistic family approach [21], TWG members expressed concern that the study did not include YPLHIV and yet YPLHIV had some of the lowest ARV adherence rates in the country. The investigators responded by expanding the intervention to cover YPLHIV, but the study budget did not permit them to evaluate YPLHIV outcomes. They gave this feedback at the next TWG meeting.

During data interpretation meetings, when SOAR researchers present study results, stakeholders ask clarifying questions, how study questions were asked; and they also provide context to explain the findings and their program/policy implications and sometimes ask for additional analyses to be performed. SOAR researchers respond by analyzing and presenting this data at subsequent engagements. Fostering this kind of partnership can generate a thorough understanding of study findings and a mutual accountability that ensures stakeholders follow up on promises they make to researchers such as garnering additional support for the study from appropriate agencies.

To ensure continued stakeholder engagement, SOAR’s researchers use the *Stakeholder Engagement Matrix,* from the RU guide, as a living tool in which they record both what has been achieved and what is planned in engaging stakeholders and they update it every 6 months and include it in their semi-annual report to SOAR management.

### Stakeholder Capacity and Skills Matter

The need for a strong capacity of research users to acquire, assess, adapt, and apply research evidence has been emphasized [5]. During scouting visits, researchers should assess the capacity of stakeholders and in-country research colleagues and develop a strategy for mentoring of upcoming researchers and stakeholders during the life of the study [8]. SOAR’s approach to capacity strengthening (CS) is multi-pronged, including SOAR researchers working side by side with in-country researchers and stakeholders, conducting regional CS workshops, and a small grants’ initiative that funds research or RU projects designed by upcoming researchers and nested in the main SOAR studies. SOAR’s team of investigators for each study includes local government officials and non-government officials as well as international scientists who learn from each other and share experiences in research and stakeholder engagement; and the investigators work side by side with key local stakeholders throughout the life of the study.

One of the key assignments of SOAR’s RU Advisor is to plan and conduct CS activities. In February 2017, SOAR conducted a CS workshop for in-country researchers collaborating on studies in 12 countries, and each researcher was joined by a study stakeholder from a governmental or nongovernmental institution responsible for program or policy implementation [22]. Following this workshop, SOAR advertised a competitive small grants initiative, out of which nine successful applicants received grants (up to $10,000) to apply the skills they learned in secondary data analysis, results utilization, and knowledge translation. In May 2018, SOAR convened a follow-up workshop to strengthen the skills of the small grants’ applicants through mentor–mentee relationships using mentors who are successful African researchers [23].

### Making Data Readily Available and in Digestible Formats is Crucial

It is not uncommon for stakeholders to be unaware of research conducted in their country until they come across it at an international conference or in a journal. One of the most frequently reported barriers to evidence uptake is poor access to good quality relevant research [12].

SOAR researchers are sharing preliminary results with stakeholders, thus giving opportunity to stakeholders to use these data to strengthen their programs while the studies are ongoing. This makes RU a virtuous cycle that generates demand for data—mimicking the well-known CQI [19] and CLA [20] strategies.

To enable stakeholders to access interim study findings, SOAR conducts data interpretation meetings to present these findings and discuss their meanings. Results briefs are produced in user-friendly formats highlighting key findings and programmatic implications for busy decisionmakers. Some stakeholders have utilized SOAR study data to improve programs even before the final study findings are published. In a study in South Africa on tracing contacts of PLHIV diagnosed with tuberculosis, the researchers shared tracing data with clinic managers on a quarterly basis [24]. Researchers reported that one of the clinic managers reacted to preliminary positive results by training CHW in contact tracing resulting in improved contact tracing rates the next quarter. In another South African study, on provider-initiated testing and counseling, researchers presented formative findings highlighting performance gaps to clinic managers who reacted by improving service delivery by offering HIV testing to clients during queuing periods prior to seeing a clinician, taking advantage of otherwise wasted time [25].

### Knowledge Translation (KT) Approach Strengthens Relations and Dialogue

A key barrier to RU is often the lack of dialogue between researchers and decision makers. To address this barrier on a sustainable basis we recommend a KT approach, defined as a process that focuses on building trust and dialogue among researchers, policy makers, and other research users. A KT process is usually led by “trustworthy, highly connected, and credible experts, intermediaries who excel in the worlds of both research and policy” [8].

Using the small grants program mentioned above, SOAR’s RU Advisor is working with study team members who are familiar with both the research and stakeholder communities in their countries to conduct KT activities. In a KT small grant activity, in Cameroon, stemming from SOAR’s study pilot testing an updated People Living with HIV Stigma Index [26], investigators engaged a community of practice to develop a code of conduct for health workers as a means to mitigate HIV stigma and discrimination in the health care setting. In Uganda the investigators of the Stigma Index study used the KT grant to engage a community of practice linked to the Uganda AIDS Commission enabling data from the study to be used in developing the national stigma policy. In another KT grant investigators translated a HIV disclosure intervention for young people, proven efficacious in a SOAR study [27] in Uganda, to the control community used for the study.

## Discussion and Conclusion

We have illustrated how SOAR researchers meaningfully and regularly engaged a wide range of stakeholders, including government officials, donors, service providers, and service beneficiaries from study inception through implementation. Prioritization of stakeholder engagement, especially the MOH is critical to successful uptake of study results. Engagement is dependent on the strength of relationships and, while it is supposed to occur in every study, it does not always happen especially at study inception [7]. Program and policy stakeholders often prioritize service delivery issues and other competing demands or may have little confidence or interest in research. Researchers may also not have the capacity to translate research or may be too absorbed in the technicalities of conducting the research that they don’t prepare results for translation [28]. Mechanisms may also not be in place to facilitate the dialogue needed for research translation and uptake. In addition, poor “quality of relationships” and lack of “trust” may exist between researchers and stakeholders [1]. For these reasons, we consider the successful engagement of stakeholders by SOAR researchers as a key achievement.

We can attribute the successful engagement of stakeholders, at least partly, to the systematic and emphatic effort of SOAR management to ensure that RU is integrated into the research process. Key to this effort was the development of the RU guide and tools that detail a systematic process of active engagement of stakeholders while taking into consideration the context in which decision makers function. A systematic and funded process of RU has been highlighted as a factor that helps research translation by ensuring that researchers undertake research with the end in mind, and stakeholders are systematically engaged at various points of evidence production [7, 29]. To this end, SOAR designated a full time RU Advisor to support its researchers in implementing the RU process. A second factor is the emphasis by the donor, United States Agency for International Development (USAID), that RU is an important reportable result of the research project, equivalent to the conduct of high-quality research. This implied that SOAR researchers were expected to fully integrate RU in their study protocols, budgets, timelines, and in the semi-annual study progress reports that they submit to SOAR management. A third issue was the insistence by USAID and SOAR management that each SOAR study should engage at least one in-country co-principal investigator on each study. These early lessons from SOAR show that the commitment by funder, implementing organization, and end data users supported by tools and processes can be an effective intervention to spur the use of research results to improve HIV service delivery.

## Footnotes

1 An activity brief is a two-page document produced by SOAR’s communication team for each study stating the study’s objectives, methods, and proposed RU process in a concise and digestible format. SOAR provides printed copies of briefs to researchers to use to explain the study as they engage stakeholders. (http://www.projsoar.org/resources/resource-type/activity_brief/).

2 For all studies, a results brief is a short document that states the study objectives, methodology, key findings, programmatic implications, and recommendations of a study in an easily digestible format. (http://www.projsoar.org/resources/resource-type/results_brief/).
